# Prediction of Conserved Peptides of *Paracoccidioides* for Interferon-γ Release Assay: The First Step in the Development of a Lab-Based Approach for Immunological Assessment during Antifungal Therapy

**DOI:** 10.3390/jof6040379

**Published:** 2020-12-19

**Authors:** Sarah Brena Aparecida Rosa, Bárbara Guimarães Csordas, Sandra Maria do Valle Leone de Oliveira, Amanda Ribeiro dos Santos, Anamaria Mello Miranda Paniago, James Venturini

**Affiliations:** 1 Faculdade de Medicina, Universidade Federal do Mato Grosso do Sul, Campo Grande 79070-900, MS, Brazil; sarahbrena113@gmail.com (S.B.A.R.); sandrinhaleone@gmail.com (S.M.d.V.L.d.O.); amandashalar@hotmail.com (A.R.d.S.); anapaniago@yahoo.com.br (A.M.M.P.); 2 Laboratório de Biologia Molecular do Carrapato, Embrapa Gado de Corte, Empresa Brasileira de Pesquisa Agropecuária—EMBRAPA, Campo Grande 79106-550, MS, Brazil; barbara.guima.csordas@gmail.com

**Keywords:** cellular immunity, paracoccidioidomycosis, in silico prediction, MHC II, IFN-γ, *Paracoccidioides brasiliensis*, *Paracoccidioides lutzii*, interferon-gamma release assay

## Abstract

Impaired antigen-specific cell-mediated immunity (CMI) is a primary immunological disturbance observed in individuals that develop paracoccidioidomycosis (PCM) after exposure to *Paracoccidioides* spp. Restoration of *Paracoccidioides*-specific CMI is crucial to stop the antifungal treatment and avoid relapses. A convenient and specific laboratory tool to assess antigen specific CMI is required for the appropriate clinical treatment of fungal infections, in order to decrease the time of antifungal therapy. We used an interferon-γ release assay strategy, used in the diagnosis of latent tuberculosis infection, to address our aims in this study. Information on proteins secreted by two well-studied representative strains—*Paracoccidioides brasiliensis* (Pb18) and *P. lutzii* (Pb-01)—were explored using PubMed or MEDLINE. From 26 publications, 252 proteins were identified, of which 203 were similar according to the Basic Local Alignment Search Tool. This enabled a selection of conserved peptides using the MEGA software. The SignalP-5.0, TMHMM, IEDB, NetMHC II, and IFNepitope algorithms were used to identify appropriate epitopes. In our study, we predicted antigenic epitopes of *Paracoccidioides* that could bind to MHC class II and induce IFN-γ secretion. These T cell epitopes can be used in the development of a laboratory tool to monitor the CMI of patients with PCM.

## 1. Introduction

Paracoccidioidomycosis (PCM) is a systemic mycosis endemic to Latin America and is caused by fungi belonging to the genus *Paracoccidioides* [1]. Based on studies of nuclear and mitochondrial genealogy, five species of *Paracoccidioides*—*P. brasiliensis*, *P. lutzii*, *P. americana*, *P. restripiensis*, and *P. venezuelensis*—are reportedly responsible for PCM [2]. However, *P. brasiliensis* and *P. lutzii* are the primary representative species used in clinical, molecular, morphological, and immunological studies on fungi–host interplay, with the findings having implications in laboratory diagnosis [1,3].

PCM is considered a neglected tropical disease and primarily affects males aged 30–50 years. It is more prevalent among rural workers. In endemic areas, three new cases of PCM are registered per 100,000 individuals annually [4]. In Brazil, PCM was considered as the eighth major cause of mortality underlying all chronic and parasitic diseases and the first cause of mortality underlying systemic diseases [5]. The infection occurs when mycelial phase propagules are inhaled by the host. Upon infection, conidia turn into yeasts within the alveolar macrophages and multiply, thereby infecting other cells. To curb the infection, Th1 and Th17 immune responses are polarized, thereby generating subgroups of T cell cells, including T helper cells (TCD4^+^) [1]. Interferon-γ (IFN-γ) released by these TCD4^+^ cells activates macrophages to produce high amounts of reactive oxygen species, which kill the fungi [6].

The fungi can remain latent for many years or decades even after Th1 polarization [1,7]. When the fungi–host interplay is disrupted, *Paracoccidioides*-infected hosts develop PCM. In this context, the Th2/Th9 immune response is dominant, and the remaining cellular-mediated immunity (CMI) is not sufficient to restrict fungal spread [8]. Moreover, persistent systemic inflammatory response is a hallmark of PCM, often conferring deleterious effects [9,10].

Antifungal therapies involve long-term regimens. The treatment duration depends on several criteria––clinical, mycological, radiological, and immunological [1]. CMI assessment is among the primary challenges in PCM treatment. Detection of specific antibodies using double agar gel immunodiffusion (IDD) has helped clinicians in deciding the extent and duration of treatment [11,12]. However, it is important to note that IDD evaluates humoral immunity and not CMI and is limited in its sensitivity. Another factor limiting the use of IDD is that 10–40% of the patients diagnosed with PCM do not test positive before and/or during the treatment, thereby posing challenges to the decision-making process for cessation of the treatment [4]. The development of a lab-based approach to accurately assess the recovery of antigen specific CMI can help overcome this challenge. The IFN-γ release assay (IGRA) is a laboratory-based approach that has been used successfully in the immunological screening of latent tuberculosis infection (LTBI). Briefly, a small volume of whole blood is incubated with purified peptides from *Mycobacterium tuberculosis* for 24 h at 37 °C [13]. The recognition of tuberculosis antigens by primed TCD4^+^ lymphocytes from infected individuals results in the production of significant amounts of IFN-γ, the levels of which are detectable by ELISA [14]. Considering that patients with PCM present with increased circulating clone-specific TCD4^+^ levels after the recovery from CMI [1], in the present study, we predicted conserved antigenic peptides of *P. brasiliensis* and *P. lutzii* that would bind to class II MHC molecules and induce secretion of IFN-γ, using in silico prediction approaches.

## 2. Material and Methods

### 2.1. Exploration of Candidate Proteins

**Scheme 1:**
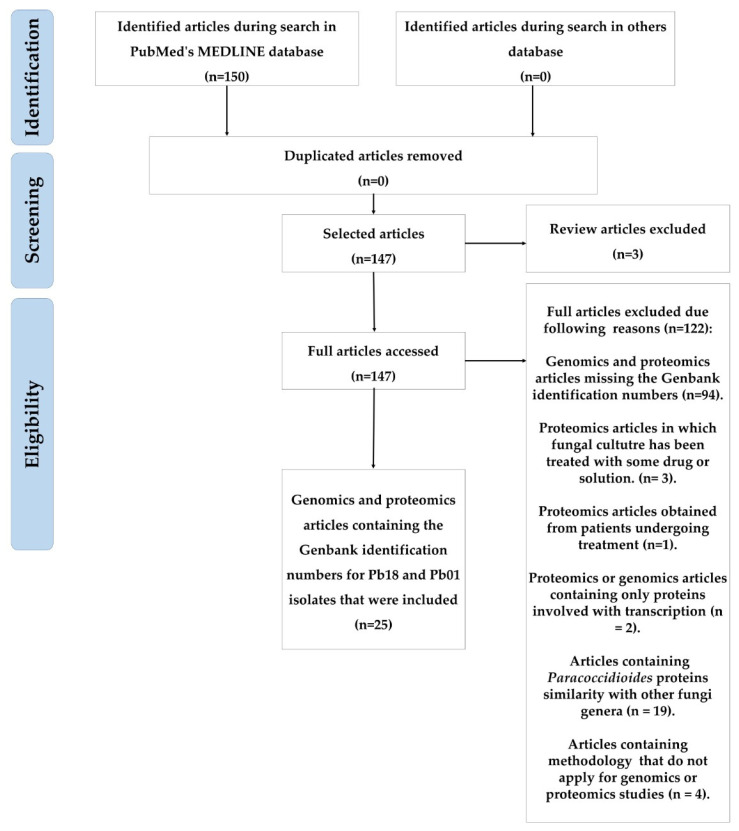
Detailed flowchart for the rapid review steps.

Conserved peptides were explored using PubMed or MEDLINE between April 2019 and December 2019. The following descriptors were used: *Paracoccidioides*, proteomics, and genomics. These words were separated with the commands AND and OR as follows: *Paracoccidioides* “[All Fields] AND (proteome * [tw] or genome * [tw])”. The results of the search included articles relevant to the proteomics and genomics of *Paracoccidioides* species. Only those articles containing data on *P. brasiliensis* (strain Pb18) and *P. lutzii* (strain Pb01) were included; only proteins obtained via genomic (molecular phylogeny, polymerase chain reaction, transcriptomics, and secretomics) and proteomic (NanoUPLC–MS or LC–MS/MS) methods were included in the study. Review papers with descriptions of fungi and methodological comparisons were excluded. Two authors (SBAR and BGCC) read the summaries of all the included articles and duplicates and those that were not in accordance with the inclusion criteria. Finally, 147 articles were selected and screened in detail, as indicated in Scheme 1.

### 2.2. Bioinformatics Prediction Programs 

**Scheme 2:**
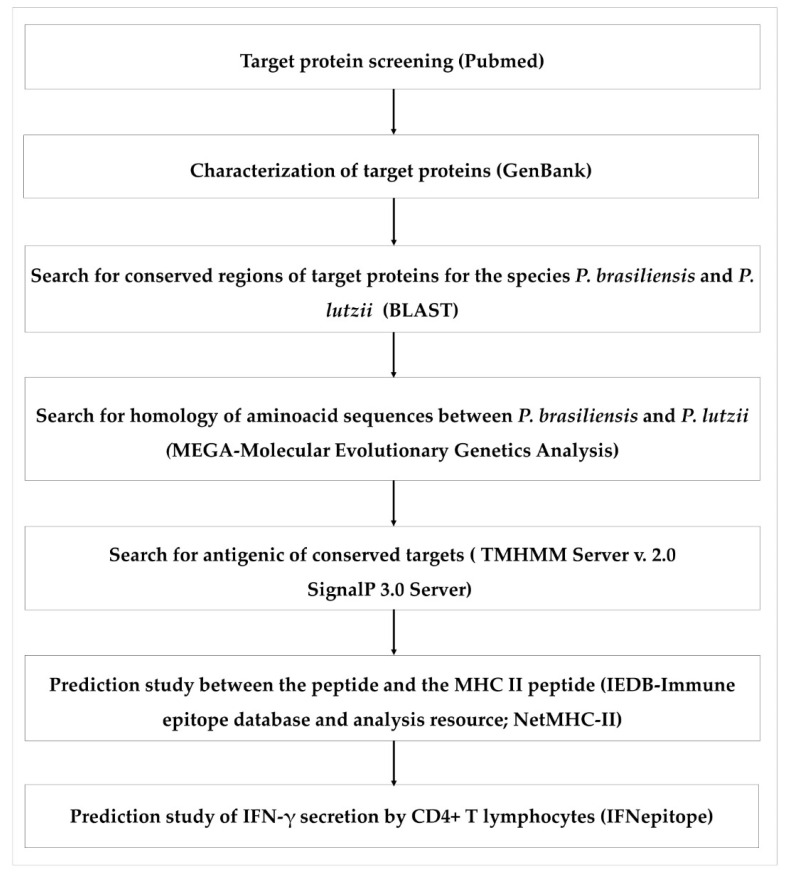
Flowchart of the experimental design.

Screening for peptides of *P. brasiliensis* and *P. lutzii* that would cause IFN-γ release in IGRA involved the following steps: 1. target protein screening, 2. characterization of target proteins based on conserved region (CDD)-related proteins associated with energy production, cellular respiration, and immune defense, 3. search for conserved regions of the target proteins, 4. search for homology of amino acid sequences between the two species, 5. search for conserved antigenic regions, 6. prediction of immunogenicity of the binding peptide to class II MHC molecule, and 7. prediction of IFN-γ secreted by T cells. The study design has been depicted in Scheme 2.

### 2.3. Investigation of Amino Acid Sequences

The first step comprised identification of upregulated proteins secreted in the yeast phase using GenBank (Available online: https://www.ncbi.nlm.nig.gov/pubmed (accessed on 02 April 2019). The CDD5(conserved domain) was assessed to elucidate the protein classification and functions. Proteins with multiple functions and nuclear activity and G-type glycoproteins (GPIs) were excluded.

### 2.4. Alignment of the Amino Acid Sequences of P. brasiliensis and P. lutzii

*P. brasiliensis* and *P. lutzii* protein sequences were aligned using the basic local alignment search tool (BLAST) [15] and downloaded in FASTA format from GenBank. The alignment between the epitopes of *P. brasiliensis* and *P. lutzii* was assessed using the protein–protein function (Available online: https://blast.ncbi.nlm.nih.gov/Blast.cgi ((accessed on 02 May 2019)). This platform compares the amino acid sequences against reference sequences in GenBank using the following criteria: Query (average converted into a percentage of the sequences in GenBank), E-value (respective significance value of *p* < 0.01) and identity (intended for the percentage of similarity between the sequences).

### 2.5. Identification of Conserved Peptides from Protein Sequences of P. brasiliensis and P. lutzii

To identify conserved peptides, the MEGA software v.7.0 (Molecular Evolutionary Genetics Analysis) was used [16]. The tool showed variable and conserved regions along the sequences in pairs obtained in the alignment. From the conserved regions, sequences that were 15 amino acids in length (peptides) were selected for antigenicity and immunogenicity analyses.

### 2.6. Prediction of Antigenic and Immunogenic T Cell Epitope

Antigenicity prediction is essential for proper peptide synthesis because it indicates whether a peptide can bind the MHC class II molecule and form an antigen complex that is recognized by T cell receptors [17]. To determine which peptides would bind the MHC class II molecule, two predictors—signal webserver v.3.0 and TMHMM webserver v.2.0—were used to indicate signal peptide cleavage sites, with a cutting bridge <0.4 on the D axis [18]. Thus, the selected peptides would remain intact without undergoing cleavage, thereby integrating the MHC molecule. TMHMM webserver v.2.0 analysis revealed that the peptides in the host cell were in the extracellular portion of the cell membrane. A threshold of >1 was considered for extracellular peptide regions [19]. To predict immunogenicity, we used two commonly accessed IEDB data sets (Free Epitope Database and Prediction; available online: https://www.iedb.org/ (accessed on 02 May 2019) and NetMHCII webserver v.2.3 (Available online: http://www.cbs.dtu.dk/services/NetMHCII (accessed on 02 May 2019). IEDB is a data bank that integrates different algorithms for prediction of the binding affinity between peptides and HLAs. Results were obtained via NetMHCpanII, NNAlign, SMMalign, Storniolo, and Consensus methods, all based on the <1% rank for strong binders [20]. In this study, epitopes were predicted for the seven subsets of HLA alleles existing in the overall global and Brazilian populations (HLA-DP, HLA-DQ, and HLA-DR). NetMHC II webserver v. 2.3 is an algorithm that can predict the binding affinity between peptides and HLAs (HLA-DR, HLA-DP, and HLA-DQ). However, this tool is based on the percentage rank and affinity, which is calculated using the formula 1-log(IC_50_nM)/log(50.000). Both methods classify potential ligands with values of <1 [21].

### 2.7. Prediction of IFN-γ-inducing MHC Class II Binders

The IFNepitope algorithm was used to predict if the selected epitopes would induce secretion of IFN-γ [16]. Antigenic regions were explored to confirm the ability of these epitopes to induce IFN-γ secretion via MSV, classifying inducers as a positive and non-inducers as a negative. Positive epitopes presented with a score of approximately 0.0.

### 2.8. Validation Screening

To validate our results, the ESAT-6 protein was used as a model. This protein was used in commercial IGRA tests for LTBI and represents strong immunogenic epitopes, which are recognized during tuberculosis infection [22]. The ESAT-6 sequence was downloaded (GenBank: ABD98021.1) and submitted for analysis using the following algorithms: Signal P, TMHMM, NetMHC II, IEDB, and IFNepitope. These data are shown in (Figure S1).

## 3. Results

### 3.1. Selected Articles and Protein Inclusion Criteria

Of the 150 articles, 25 containing information on the identity of the extracted amino acid sequences in GenBank corresponding to *P. brasiliensis* and *P. lutzii* were included; these included articles that described yeast proteins obtained from preparations of fungal culture, plasma, or peripheral blood mononuclear cells without the intervention of any drugs. Upregulated proteins associated with the fungal cell wall structure, respiration, defense, virulence, and induction of the immune response were included. The sequence identities were searched for in the introduction, methodology, results, or discussions of the articles listed in Table 1.

### 3.2. Analysis of Proteins Based on Their Conserved Domain Database

First, 252 proteins were classified according to their family based on data from the conserved domain database (CDD); 39 proteins related to nuclear activity and with multiple functions were excluded. Similarly, G-type proteins, such as GPI glyceraldehyde aldolase, fructose-1,6-bisphosphate aldolase, glyceraldehyde-3-phosphate, and GTPases, were excluded because the peptide ligands could not dissociate from the host intracellular membrane; these would not be consequently expressed on the cell membrane associated with the MHC molecule.

### 3.3. Similarity Analysis between the Proteins Selected for P. brasiliensis and P. lutzii

A total of 213 amino acid sequences were submitted for BLAST analysis. The sequences were considered similar if they presented an E-value of 0.0, query of 97–100%, and identity of 90–100%. Of these, 13 proteins had no similarity in alignment for any of the two species and for aldehyde dehydrogenase (PADG_01174), L-threonine-3-dehydrogenase (PAAG_00966), 1,2 dihydroxyl-3-keto-methylopentene-dioxygenase (gI226294753), 2.5 diceto-D-gluconic reductase A (giI295663891), glutamate reductase (giI295664022), aldehyde dehydrogenase (PAAG_05249), enolase (ABQ45367), aminobutyrate aminotransferase (PADG_02214), and PADG_34 proteases. 

Another 63 proteins were excluded for not fulfilling any of the similarity criteria. A total of 127 proteins were included based on their query score; however, despite having an E-value cutoff equivalent, two proteins––aqualysin (PADG_04168) and 1,3 β-gluconase (PADG_07461)––exhibited <90% identity and were excluded. A total of 125 antigen sequences were selected for further analysis (Table 2).

SignalP identified 19 proteins with signal peptides in most of the *P. brasiliensis* and *P. lutzii* sequences. However, of the nineteen proteins, the TMHMM server identified only six sequences that exhibited transmembrane propellers. Nevertheless, these proteins were included in the peptide analyses as they revealed extracellular regions in most sequences. In this study, only seven sequences were intracellular and were excluded. Immunogenic analysis by NetMHC and IEDB of the sequences indicated that optimal epitopes were present in the following proteins—interalpha trypsin (PADG_06178), chitin synthase class VII (>ABV31248.1), peroxisomal hydratase–dehydrogenase–epimerase (PADG_08651), and phosphoenolpyruvate carboxykinase (PAAG_08203).

**Figure 1:**
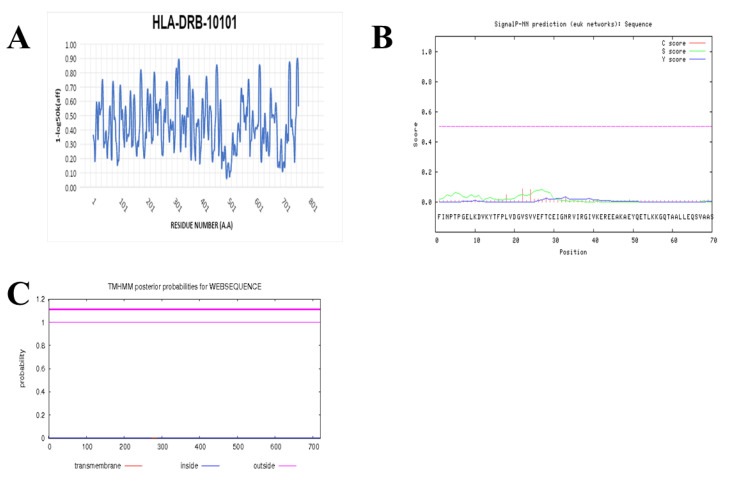
Interaction analysis of interalpha trypsin (PADG_06178) by integrating different algorithms. (**A**). Signal peptide prediction (signal peptide). (**B**). Prediction of transmembrane regions (TMHMM). (**C**). Prediction of epitopic regions for MHC II (NetMHCII).

NetMHC II analysis of interalpha trypsin identified the HLA-DRB10101 alleles related to MHC epitope 1 as the binding partner—MSAFSRMTASLGFSK (15 amino acids, amino acid 510–526). The results showed a percentage rank of 0.07 and an affinity calculation of 0.9, thereby indicating this epitope as the strongest ligand in the group. For epitope 1, no signal peptide was found by the SignalP algorithm, whereas TMHMM located it outside the cell membrane (Figure 1).

**Figure 2:**
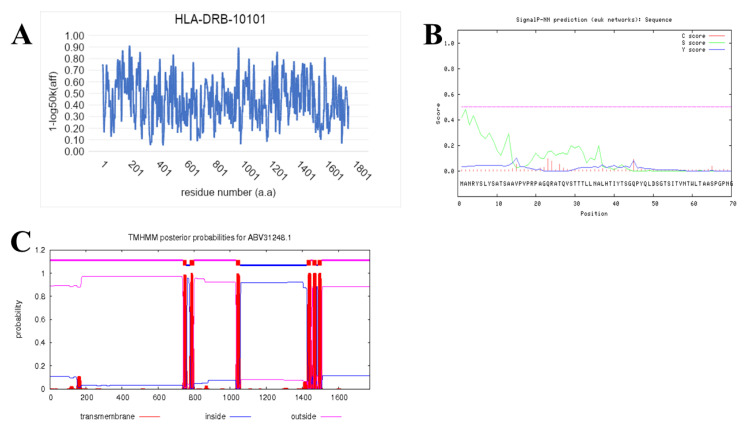
Analysis of the interaction of class VI protein chitin synthase (>ABV.31248.1) by integrating different algorithms. (**A**) Signal peptide prediction (Signal peptide). (**B**). Prediction of transmembrane regions (TMHMM). (**C**). Prediction of epitopic regions for MHC II (NetMHCII).

For class VI chitin synthase (ABV31248.1), NetMHC II identified alleles corresponding to MHC (HLA-DRB10101) for peptide 2—FDFYYLLTSASTPA (15 amino acids, amino acid 193–208)—considering the rank percentage of 0.05 and affinity calculation of 0.9. For epitope 2, no signal peptide was found by SignalP and TMHMM in the extracellular portion of the membrane (Figure 2). 

**Figure 3:**
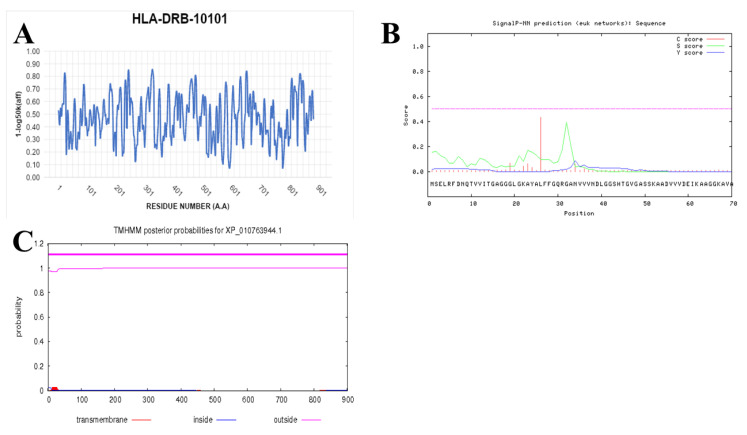
Analysis of the interaction of the peroxisomal protein hydratase dehydrogenase epimerase (GenBank: PADG_06851) by integrating different algorithms. (**A**) Signal peptide prediction (signal peptide). (**B**). Prediction of transmembrane regions (TMHMM). (**C**). Prediction of MHC II epitopes (NetMHCII).

For peroxisomal protein hydratase (PADG_06851), NetMHC II identified alleles corresponding to the MHC (HLA-DRB10101) for epitope 3—RAYALLFSKLGAAVV (15 amino acids, amino acid 326–341)—with a rank percentage of 0.5 and affinity calculation of 0.8. For epitope 3, no signal peptide was found with the SignalP algorithm, and it was located in the outer part of the membrane (Figure 3).

**Figure 4:**
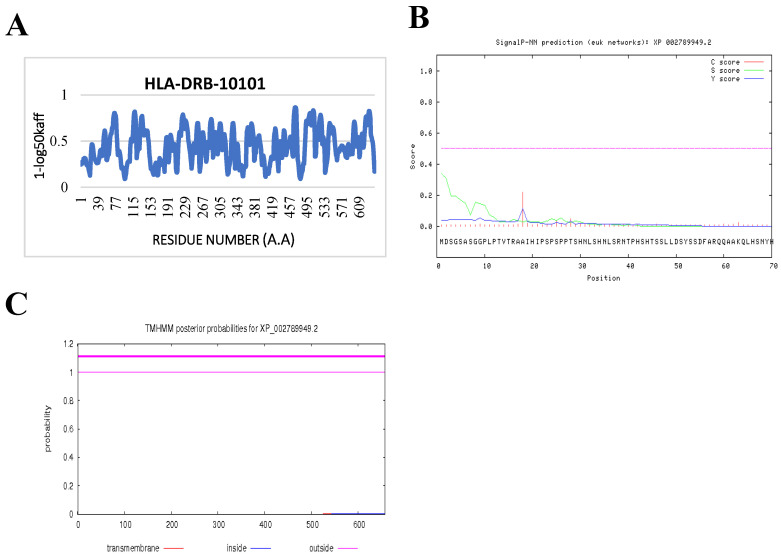
Analysis of the interaction of the phosphoenolpyruvate carboxy kinase protein (PAAG_08203) by integrating different algorithms. (**A**) Signal peptide prediction (signal peptide). (**B**). Prediction of transmembrane regions (TMHMM). (**C**). Prediction of MHC II epitopes (NetMHCII).

For phosphoenolpyruvate carboxykinase (PAAG_08203), NetMHC II identified alleles corresponding to MHC (HLA-DRB10101) for epitope 5—ERVSIIANPAVASLY (15 amino acids, amino acid 118–132)—with a rank percentage of 1.6 and affinity calculation of 7.5. For epitope 4, no signal peptide was found with the SignalP algorithm, and it was located on the outside of the cell membrane (Figure 4).

Based on our findings, an in-depth analysis of the high-frequency peptides MSAFSRMTASGFSK (PADG_06178), FVFYYLLTSASTPA (ABV.31248.1), RAYALLFSKLGAAVV (PADG_08651), and ERVSIIANPAVASLY (PAAG_08203) was performed (Table 3).

### 3.4. Prediction of IFN-γ inducing MHC Class II Binders 

To determine which of the four epitopes could lead to the best, possible, abundant secretion of IFN-γ, three different analyses were performed using IFNepitope. To confirm the results, we used ESAT-6 as a control, as shown in Table 4.

## 4. Discussion

In the present study, in silico analyses allowed the prediction of immunogenic epitopes from *P. brasiliensis* and *P. lutzii* antigens. In this prediction, four peptides with fifteen amino acids restricted to the HLA class II molecule were identified as the optimal epitopes in the recognition of T cells. 

These peptides were obtained from interalpha trypsin, chitin synthase, peroxisomal hydratase–dehydrogenase–epimerase, and phosphoenolpyruvate carboxykinase. In general, these proteins are produced when the fungus undergoes a mechanism of adaptation to the intracellular environment, in order to survive in a latent state [28,32,33,41,48]. The low-oxygen environment allows the fungus to survive via metabolic adaptation, thereby enabling the fungus to evade intracellular defense mechanisms while remaining protected by the endosome. To stay into the endosome, the fungus inhibits the phagolysosome maturation, lyses the host cell, detoxifies oxidative or nitrosative reagents, and uses different metabolic pathways to obtain energy from the available nutrients [26,49,50]. This mechanism is characterized by interactions between the fungus and host molecules and with the components of the cellular matrix, thereby causing delays in immune response induction [51]. Despite efforts required for fungal evasion, infected macrophages can act as antigen-presenting cells APCs, and can process and present antigens to TCD4^+^ cells. Recognition promotes the differentiation of TCD4^+^ cells into auxiliary T (Th1) cells, which when activated secrete IFN-γ, further activating the microbicidal function of alveolar macrophages and stimulating the presentation of antigens at the infected sites [52].

The alignment of protein sequences using BLAST made it possible to acquire data on conserved peptides of *P. brasiliensis* and *P. lutzii* (Table 2). The aligned sequences when compared with GenBank sequences exhibited high similarity, thereby indicating that a conserved peptide could be identified. As *P. brasiliensis* and *P. lutzii* are detected at a higher frequency in Brazil [4] compared to that detected in the remaining regions of the world, only proteins of *P. brasiliensis* and *P. lutzii* were prioritized. As recommended, the alignment of protein sequences was assessed by checking the codons of the genome between the two species (ClustalW). This allowed us to select conserved peptides and to confirm the conservation of immunogenic epitopes predicted by the immunogenicity prediction analysis tools. Next, antigenicity and immunogenicity analyses were performed for these conserved peptide regions with reference to 125 genomic protein sequences. Thus, it was possible to identify the best HLA class II epitopes [53].

Four strong candidate peptides for stimulation of the in vitro cellular immune response were found––MSAFSRMTASLGFSK, FDVFYYLLTSASTPA, RAYALLFSKLGAAVV, and ERVSIIANPAVASLY. MSAFSRMTASLGFSK has been identified in interalpha trypsin peptide. This protein stimulates cellular differentiation [32]. High levels of trypsin have been observed in the sputum of patients with pulmonary cystic fibrosis. This is because the protein induced mucus hypersecretion and promoted the leukocyte recruitment to the inflammation site [54].

FDVFYYLLTSASTPA is part of the chitin synthase protein sequence, which forms α-glucan, a fungal cell wall layer in yeast. Studies have shown that high amounts of chitin synthase secreted by the fungus via proteolytic enzymes of the phagolysosome are used to reconstruct and maintain the cell wall [45,55,56]. Recently, this immunomodulatory activity of chitin synthase was demonstrated in the maturation of dendritic cells infected by *P. brasiliensis,* in vitro [57].

RAYALLFSKLGAAVV is a peroxisomal hydratase–dehydrogenase–epimerase peptide and studies have shown that the secretion of this protein is increased under stress conditions in fungal cultures and phagocytes infected by *Paracoccidioides*. It inhibits oxidative reactions and detoxifies molecules released by the lysosome [26,29,51].

ERVSIIANPAVASLY is a phosphoenolpyruvate carboxykinase peptide abundant in macrophages infected by *Paracoccidioides* [30]. Immunogenic epitopes of this antigen have already been identified in *Leishmania major* and *L. donovani* and have been used to develop a recombinant vaccine for leishmaniases. In animal models, this vaccine has shown promising results since the phosphoenolpyruvate epitopes activate TCD4^+^ lymphocytes, thereby promoting clonal expansion and increasing IFN-γ secretion [58].

In antigenicity analysis, proteins with a peptide signal at the beginning of the sequence or those lacking the signal exhibited higher affinity for antigenic epitopes. To determine this, we used two predictors—SignalP and TMHMM. SignalP revealed which peptides would be located in extracellular regions of proteins. The obtained results were confirmed using TMHMM, which showed that the peptides were located on the host cell membrane surface, thereby indicating an association with MHC. Both predictors have been used in studies analyzing proteins secreted by *Fusarium oxysporum* and *Alternaria brassicicola* for elucidation of proteins that are intracellular molecules and are involved in the pathogenesis of fungal infections [59].

Our four candidate peptides were extracellular molecules; in contrast, seven proteins were excluded because they presented signal peptides and cleavage sites in almost all peptide regions, thereby indicating an association with intracellular catalytic mechanisms. This may increase the costs associated with the production of these peptides as they could be cleaved during in vitro stimulation in blood monocytes. These analyses were possible because SignalP, in particular, is a predictor that classifies proteins as secretory or non-secretory in addition to indicating cleavage sites in the image. Thus, proteins that will be transported outside the cell and those that are localized inside the cytoplasm can be identified [60].

Although some proteins having a GPI anchor (e.g., glyceraldehyde dehydrogenase and fructose 1.6 bisphosphate) show no secretory signals, the presence of signal peptide in the C terminal chain has been considered [53]. This signal peptide was confirmed by the transmembrane helix analysis (TMHMM); therefore, although some proteins were antigenic, they were excluded as the peptides could not disintegrate from the membrane and cross the host cell membrane.

The results of the predictions performed by the NetMHCII and IEDB algorithms were promising as the epitopes with the highest binding affinity demonstrated values close to 0. The analysis of seven different subsets of HLA existing in the population showed that the four peptides identified exhibited excellent affinity, considering their IC50 and NNAlign rank [61].

Furthermore, IEDB analysis showed that among the four peptides, Pep_H1 MSAFSRMTASLGFSK had a high binding affinity coverage for HLA-DRB1 * 07: 01. Additionally, two peptides showed satisfactory affinity for the HLA-DRB1 * 15: 01 allele––RAYALLFSKLGAAVV and ERVSIIANPAVASLY. Thus, based on the knowledge that the most frequent alleles in the Brazilian population are HLA-DRB1 * 15: 01, HLA-DRB1 * 07: 01, and HLA-DRB1 * 03: 01, the four peptides found exhibited appropriate coverage for the Brazilian population [62,63,64].

To compare our results, we used the ESAT-6 protein of *M. tuberculosis* that was used as a positive control in the IGRA. In NetMHCII, ESAT identified two strong binding epitopes of rank <50% (epitope 1, QWNFAGIEAAASAIQ; epitope 2, WFAIEAAASAIQG). The IEDB showed greater binding affinity for HLA-DRB1 * 07: 01, HLA-DRB1 * 15: 01, HLA-DRB5 * 01: 01, HLA-DRB4 * 01: 01, HLA-DRB3 * 01: 01, and HLA-DRB1 * 03: 01 in the global population and to HLA-DRB1 * 07: 01 and HLA-DRB1 * 03: 01 in the Brazilian population. However, in the antigenicity analysis, the peptides QWNFAGIEAAASAIQ and WFAIEAAASAIQG did not show a peptide signal in SignalP and were located outside the host cell membrane as per TMHMM. Comparing the epitopes of ESAT-6 with the ones we found, the highest coverage of class II MHC was noted for the highest-class coverage percentage of 0% and <50%.

Epitope 4 induced the highest IFN-γ secretion in silico, via MHC class II. This result was similar to that found for the ESAT-6 epitope. Epitopes 2 and 3 have been shown to induce greater production of IFN-γ. Only epitope 2 did not show any relation with IFN-γ. We used the IFNepitope algorithm to confirm the induction of cytokines such IL-4 by epitope 2. However, for this purpose, it is essential to utilize the support vector machine (SMV). Furthermore, the relationship of IL-4 shows the involvement of these antigenic regions with the humoral immune response [20] and may be used in other contexts. In summary, the results show that epitope 4––ERVSIIANPAVASLY––can be most easily synthesized and incorporated into the IGRA laboratory tool for diagnosis and monitoring of PCM.

## 5. Conclusions

The present study predicted, in silico, four conserved epitopes present in *P. brasiliensis* and *P. lutzii* with potential for in vitro stimulation of T lymphocytes. The epitopes demonstrated high affinity by human allelic subsets in the Brazilian population. The epitope ERVSIIANPAVASLY showed the best performance in the induction of INF-ɤ, as compared to the epitope of ESAT-6 used in tests for IGRA. Therefore, the epitope identified herein can be used in the development of an IGRA for PCM, which, in combination with clinical assessments, can assist in the clinical follow-up of patients with PCM.

## Figures and Tables

**Table 1:** Papers selected in the rapid review.

Study, Year (Reference)	Study of Methodology	Upregulated Proteins
Alegre et al. [23] 2014	Genome	Glycosyl hydrolase.
Do Amaral et al. [24] 2019	LC–MS/MS	Ligand RNA, fructose bisphosphate aldolase, nucleic acid ligand, phosphoglycerate kinase, perixosomal catalase.
Araújo et al. [25] 2017	NanoUPLC–MS	Shock protein SSB1, glucan synthase.
Araújo et al. [26] 2019	NanoUPLC–MS	Gp 43, pyruvate dehydrogenase, ATP-citrate synthase, succinyl CoA ligase, Shock protein (Hsp 90, HPs 70, Hps 88, Hps 30, Hsp 7, Hsp 70, Hsp 75), alcohol dehydrogenase, pyruvate dehydrogenase. Cap 20, progesterone ligand, regulatory myosin cdc 4, mitochondrial perodoxin PRX1, DNA ligand, shock protein 88, serine phosphatase, threonine, protein F beta acin, enolase, triosphosphate isomerase, carbonic anhydrase, vacuolar protease 4, perodoxin, acetamidase.
Baeza, et al. [27] 2017	NanoUPLC–MS	GlucosaminE-6-phosphate-deaminase, phosphoacetilglucosamina mutase, isocitrate mutase, isocitrate liase, malate synthase, 3-hydroxybutyryl-CoA dehydrogenase, 3-Cetoacyl-CoA thiolase, Acyl-CoA dehydrogenase, Acyl-Coenzyme A oxidase, Enoyl-CoA hydratase, short-chain dehydrogenase, carnitine, adenosylomocysteinase, threonine dehydrogenase, methylcrotoyl-CoA carboxilase beta, methylmalonate semialdehyde dehydrogenase, catalase.
Castilho et al. [28] 2014	LC–MS/MS	RAC, vacuolar protease, 26 S regulatory subunit, palmitoyl thioesterase, protein associated with a pathogenesis. Cap 20, ligante de progesterona, regulatory myosin cdc 4, mitochondrial perodoxin PRX1, DNA ligand, shock protein 88, phosphatase serine treonine, actin F protein beta, enolase, triophosphate isomerase, carbonic anhydrase, vacuolar protease 4, perodoxin, acetamidase.
Chaves et al. [29] 2019	NanoUPLC–MS	Thioredoxin, superoxide dismutase (SOD Fe+, SOD Cu +), serina protease, mitochondrial acetonitate hydratase, histidin kinase, perixossomal hydratase, shock protein (Hsp 90, Hsp 88, Hsp 70, Hsp 30).
De Curcio et al. [30] 2017	NanoUPLC–MS	Carrier ATP/ADP, Plasma ATPpase, YOP1 protein, phosphate mitochondrial, osmosensor protein, ologossacaryl transferase, alpha-1,2-mannosyltransferase KTR1, dolichol-phosphato aminotransferase, phosphoinositide phosphate, oxoisovalerate dehydrogenase beta, serine-3-dehydrogenase.
Chaves et al. [31] 2017	NanoUPLC–MS	Carrier ATP/ADP, plasma ATPase, YOP1 protein, mitochondrial phosphate, osmosis protein, oligosaccaccharyl transferase, alpha-1.2-mannosyltransferase KTR1, dolichol-phosphate aminotransferase, phosphoinositide phosphate, oxoisovalerate dehydrogenase 3
Desjardins et al. [32] 2011	Genome	Chitin synthase, Shock protein (Hsp 60, Hsp, 88, Hsp 90, Hsp 7, Hsp 70), mycoserosic acid synthase, acyl transferase, Tioredoxina, perixosomal hydratase, histidine kinase, MATA_HMG-box, Interalpha-trypsin, methyltransferase, N-acetyltransferase, peptidase, transketolase, glucoamylase, PADG_11448, PADG_01788.
De Oliveira, et al. [33] 2018	LC–MS/MS	Phosphoglycerato kinase, glucose-6-phosphasto isomerase, phosphomannomutase, triosephosphate isomerase, homogentified 1,2-dioxigenase, alpha-ATPase subunit, 12-oxophytodienoate reductase, PAAG_00340, ATP citrate synthase.
Lima et al. [34] 2014	NanoUPLC–MS	4-hydroxylphenolpyruvate dehydrogenase, alanine-glyoxylate aminotransferase, cysteine dioxygenase, aspartate aminotransferase, choline dehydrogenase, glutamate decarboxylase, methylcrotonoyl-CoA carboxylase, sorbitol SOU2, inosine-dehydrogenase-5-monophosphate, fumarylacetoacetate hydrolase, WD40, ATPase, calnexin, complet T subunit T epsilon, tetraclycine.
Munhoz, et al. [35] 2016	Genome	3-hydroxyantaranilate 3,4 dioxygenase, 1,3 glucanase, BUD 32 kinase, glucan 1,3 glucosidase, aminopeptidase M18, serine protease, LOL, cation efflux, RING, phosphatidyl inositol, PADG_00954, thioredoxin, amino acid permease, acyl-CoA dehydrogenase, 3-ketoacyl reductase, transferrin peptidase.
Oliveira et al. [36] 2016	NanoUPLC–MS	Epsilon protein, Rad24, ARF GTPase.
Parente, et al. [37] 2011	LC–MS/MS	2-nitropropane dioxygenase, hydroxyacilglutathione hydrolase, L-threonine-3-dehydrogenase, spermidine synthase, glucokinase, pyruvate dehydrogenase, component X protein, nucleoside-diphosphate epimerase, thioredoxin, pentafunctional AROM, adenisulfate kinase, cytochromo c, anikirina, ubiquitin E1, citocromo C, phosophoglycerate kinase, D-hexose-6-phosphate epimerase, hydrolipoyl dehydrogenase, carnitine O acetyltransferase, perixosomal catalase.
Parente, et al. [38] 2013	MALDI–MS/MS	Thioredoxin, protein Y20, aldehyde dehydrogenase, shock protein (Hsp 30, Hsp 30, Hsp 70, Hsp 88, SSB1), malate dehydrogenase, methylcitrate synthase, co-chaperone mitochondrial GrpE, 6-phosphogluconolactonase, xanthine phosophoribosyl transferase, aldehyde dehydrogenase, aminoacyl-tRNA-synthase, oxidoreductase, oxalocrotonate tautomerase, metalloproteins, formamidase.
Parente-Rocha et al. [39] 2015	Nano-ESI-UPLC–MS	Phosphoglucomutase, hydrolipoyl-dehydrogenase, succinate dehydrogenase, alanine aminotransferase, aspartate aminotransferase, 4-hydroxyfenylpyruvate dioxigenase, vacuolar aminopeptidase, carboxypeptidase Y, aspartyl protease, protepian Y20, monitiol glutaredoxin, thioredoxin, cytochromo c peroxidase, Superoxido dismutase Cu/Zn, glucosamine-frutose-6-phosphato, ATP synthase F1F0, NADP glutamato dehydrogenase, gama glutamyltranspeptidase.
Pigosso et al. [40] 2013	MALDI- MS/MS	L-threonine-3-dehydrogenase, 1,2 diyidroxi-3-keto-5-methyllopententene-dioxigenase, 2,5-diceto-D-gluconic acid reductase A, glutathione reductase, 27 KDa, glycoprotein mitochondrial, DNA ligand, corusmate mutase, formamidase fator-1-alpha, phosphoenolpiruvato carboxikinase, 12-oxophitodienate reductase, citrate synthase, gi295666522.
Pigosso et al. [41] 2017	NanoUPLC–MS	Formamidase, carnityl-CoA dehydratase, acil-CoA dehydratase, GABA permease, integral membrane protein, 12-oxophytodinoate reductase, ABB effux, dienetaelone hydrolase, cysteine protease PalB, xanthine dehydrogenase, phosphotransferase, PADG_00675, treonina dehydrogenase, glioxilase, acil-CoA dehydrogenase, metallohydrolase.
Rezende et al. [42] 2011	MALDI- MS/MS	Mitochondrial peroxiredoxin, mannitol-1-phosphato-5-dehydrogenase, aldehyde dehydrogenase, ciclofilin, cofilin, protein G, trompomyosin, hydrolase, phosphoglycerate kinase, cobalamin, phosphoglucomutase, acetylmomoserine(tiol)-liase.
Tamayo et al. [43] 2016	Genoma	Superoxide dismutase (SOD, SOD Cu/Zn).
Tashima et al. [44] 2015	LC–MS/MS	Alpha -tubulin.
Tomazetti, et al. [45] 2019	LC–MS/MS	1,6 Glyceradeído-3-phosphate-dehydrogenase, Aqualysin, Fructose-1,6-biphosphate aldolase, enolase, Superoxide dismutase Fe+, thioredoxin reductase, succinil-CoA ligase subunidade beta, methyl-2-citrate-synthase, cytochromo c1 mitochondrial heme, Hsp 30, Hsp 70, cytocromo C peroxidase, oxirredutase, Ras-2, methylcitrate dehydrogenase, thioredoxin, 5-aminolevulinato synthase, serine protease, RPA3, ATPase, Profilin, reductase ferric, S-adenosylmethionine dehydrogenase-dependent-2-methyltransferase beta, pyruvate dehydrogenase subunit E1 beta, dyhydrolipoamide acetyl-transferase, glucosamine-fructose-6-phosphate, GTPase RhoA, receptor endossomal Erp3, Shock protein (Hsp020, Hsp 70), perixosomal, citochromo c peroxidase, phosphoesterase, cofilin, estrictosidin synthase, destrin, RRM-Srp1p.
Tristão, et al. [46] 2015	Proteomic	Superoxide dismutase (SOD, SOD Cu/Zn, SOD cytosolic, SOD cytosolic Cu/Zn), laccase 1, L-ascorbitate oxidase, laccase IV, cupredoxin, alcohol dehydrogenase, class II aldolase, PADG_00743, mannose-6-phosphate isomerase, peptidase M1, copper carrier ATPase, heavy metal ATPase, urease, D-arabinose-1-dehydrogenase, calcium carrier, carrier CCC1, calcium carrier, iron and magnesium, cytochromo, ypt5 binding GTPase, mitochondrial porin, SEC62 protein, chpA protein, binding GTPase ypt7, chaperone, ECM33 precursor protein, extracellular matrix component, GTPase sar1, G2/M RNA ligand, iron carrier, vacuolar protein, Ras, clatrin, beta-glucosidase, endosome carrier.
Weber, et al. [47] 2012	MALDI-Q-TOF MS	Aminotransferase, fumarylacetoacetase, beta-glycosidase, glycosyl hydrolase, Grp1p, peptidyl-propyl-cis-trans-isomerase A2, disulfide isomerase Pdi1. Enolase, Hsp 10, malate dehydrogenase, serine hydroxylmethyltransferase.

**Table 2:** Alignment of amino acid sequences of *P. brasiliensis* and *P. lutzii* by the BLAST tool.

Protein	Identification	E-Value	Identity	
			Pb18	Pl 01
Carrier ADP/ATP	PAAG_08620	0.0	100%	97%
Acyl-CoA dehydrogenase	PAAG_03116	0.0	98%	100%
Acyl-CoA dehydrogenase	PADG_06805	0.0	100%	98%
Acyl CoA dehydrogenase	PADG_07604	0.0	100%	97%
Acyl CoA hydratase	PAAG_06309	0.0	97%	100%
Actin F protein subunit uptake protein	PADG_07756	0.0	100%	100%
Alcohol dehydrogenase	PADG_01174	0.0	100%	97%
Alpha-1,2 mannosyltransferase	PAAG_02462	0.0	100%	98%
Alpha-1,2 mannosyltransferase KTR1	PAAG_07238	0.0	97%	100%
Adenosillomocysteine	PADG_02859	0.0	99%	100%
Aminotransferase	PAAG_03045	0.0	100%	98%
Aminotransferase	PAAG_00053	0.0	98%	100%
ATP citrate synthase	PADG_04993	0.0	100%	98%
ATP_dependent 26S on proteasome regulation	PAAG_01926	0.0	99%	100%
Calnexin	PAAG_07037	0.0	95%	100%
Carrier ADP/ATP	PAAG_08620	0.0	100%	97%
Carrier calcium	PAAG_07762	0.0	100%	98%
Carnitil CoA dehydrogenase	PADG_05773	0.0	100%	97%
Catalase	PAAG_01454	0.0	100%	98%
Catalase	PADG_00324	0.0	98%	100%
Catalase	PAAG_01943	0.0	99%	99%
Chitin synthase VII class	ABV31248.1	0.0	98%	99%
1,3 ketoacyl-CoA lyase	PADG_07365	0.0	100%	98%
3-ketoacyl reductase	PADG_01943	0.0	100%	95%
Citrate synthase	PAAG_08075	0.0	98%	100%
Colin acetyltransferase	PADG_07023	0.0	100%	98%
Gamma subunit complex T	PAAG_07165	0.0	99%	100%
Corismate mutase	PAAG_05198	0.0	98%	100%
Dehydrogenase	PADG_07369	0.0	100%	98%
Dihydrolopiol	PAAG_03330	0.0	97%	100%
Dihydrolopiol dehydrogenase	PAAG_06494	0.0	100%	97%
Dolichol-phosphate mannosyltransferase	PAAG_01874	0.0	98%	100%
Enoyl coenzyme crontonase	PADG_01209	0.0	100%	97%
Formamidase	PAAG_03333	0.0	99%	98%
Formamidase	PADG_06490	0.0	100%	97%
Fumarylacetoacetoato hydrolase	PAAG_00869	0.0	98%	100%
Glutamyl transferase range	PADG_01479	0.0	97%	100%
Glutamate dehydrogenase	PADG_04516	0.0	100%	98%
Glucan synthase	PADG_07373	0.0	97%	100%
Glycosamine-6-phosphate deaminase	PADG_00401	0.0	100%	97%
Hydratase mitochondrial acetones	PAAG_00845	0.0	100%	99%
Hydroacylgluthatione hydrolase	PAAG_02548	0.0	98%	100%
4-Hydroxyfenylpyruvate dioxygenase	PAAG_08468	0.0	99%	100%
Hexose-6-phosphato epimerase	PADG_03243	0.0	98%	100%
Hydratase	PADG_11845	0.0	99%	100%
Hydroxyl-CoA dehydrogenase	PADG_01228	0.0	100%	98%
Hydroacylglutathione hydrolase	PAAG_02548	0.0	98%	100%
Histidin kinase	PADG_11468	0.0	100%	96%
Hypothetical protein	PAAG_02761	0.0	94%	100%
Hypothetical protein	PADG_01788	0.0	98%	100%
Hypothetical protein	PADG_01788	0.0	98%	100%
Interalpha trypsin	PADG_06178	0.0	100%	96%
Inosine-S-monophosphate dehydrogenase MD2				
Isocitrate lyase	PAAG_04542	0.0	98%	99%
Isocitrate lyase	PAAG_06951	0.0	100%	98%
3-isopropyl dehydrogenase	PAAG_05328	0.0	97%	100%
L-threonine dehydrogenase	PAAG_00966	0.0	99%	100%
Laccase	PADG_06196	0.0	100%	96%
Mallate dehydrogenase	PAAG_00053	0.0	98%	100%
Mallate synthase, glyoxysomal	PADG_04702	0.0	99%	97%
Mallate synthase, glyoxysomal	PADG_04702	0.0	99%	97%
Mannitol-1-phosphate dehydrogenase	PAAG_06473	0.0	100%	98%
Methyltransferase	PADG_01183	0.0	100%	94%
Methyltransferase	PAAG_09014	0.0	94%	100%
Methylcronotonoil-CoA carboxylase	PAAG_04103	0.0	98%	100%
Methilmalonate-semialdehyde dehydrogenase	PAAG_07036	0.0	98%	100%
Metalo hydrolase	PADG_03136	0.0	100%	97%
Mitochondrial	PAAG_05350	0.0	97%	100%
Mitochondrial acetonate hydratase	PAAG_00845	0.0	100%	99%
NADP-especific glutamate dehydrogenase	PADG_04516	0.0	98%	100%
2-nitropropane dioxygenase	PAAG_06693	0.0	98%	100%
Osmosensor	PAAG_04025	0.0	100%	97%
Oligosaccharide transferase	PAAG_04719	0.0	98%	100%
Oxoisovalerate dehydrogenase beta subunit	PAAG_01194.2	0.0	99%	100%
Oxoisovalerate dehydrogenase alpha subunit	PAAG_01310	0.0	98%	100%
Oxirreductase	PADG_06082	0.0	90%	100%
Plasma ATPase	PAAG_08082	0.0	99%	100%
Phosphatase ser/thre	PADG_03544	0.0	100%	100%
Plasma ATPase	PAAG_08082	0.0	99%	100%
Perixosomal catalase	PADG_01943	0.0	100%	95%
Perixosomal catalase	PADG_00686	0.0	100%	96%
Perixosomal hydratase	PADG_08651	0.0	100%	97%
Pyruvate dehydrogenase component E1 beta E1	PAAG_01534	0.0	98%	100%
Pyruvate dehydrogenase	PADG_00246	0.0	100%	98%
Shock protein SSVB1	PAAG_07775	0.0	98%	100%
Serine 3 dehydrogenase	PAAG_02354	0.0	97%	100%
Serine hydroxymethyltransferase	PAAG_07412	0.0	99%	100%
Sorbitol SOU2	PAAG_04184	0.0	98%	100%
Succinate dehydrogenase	PADG_06494	0.0	97%	100%
Succinate dehydrogenase	PADG_08013	0.0	100%	98%
Succinyl CoA ligase	PADG_02260	0.0	99%	100%
Shock protein 90	PADG_02785	0.0	100%	98%
Shock protein 98	PADG_00765	0.0	100%	99%
Shock protein SSVB1	PAAG_07775	0.0	98%	100%
Transferrin	PADG_00686	0.0	100%	98%
Transketolase	PADG_00246	0.0	100%	98%
Tetracycline transporter	PAAG_07990	0.0	97%	100%
Thioredoxin	PADG_03161	0.0	100%	97%
Thioredoxin	PADG_01551	0.0	100%	97%
Urease	PADG_03874	0.0	100%	97%
Urease	PADG_00954	0.0	100%	97%
Xaa-Pro aminopeptidase	PAAG_07500	0.0	100%	100%

**Table 3:** Prediction of epitopes for T helper lymphocytes of HLA class II alleles.

Pep_H1 MSAFSRMTASLGFSK (PADG_06178)			
Peptide	Allele	Method Used	Percentage Rank (%)
MSAFSRMTASLGFSK	HLA-DRB1 * 07:01	Consensus (comb.lib./smm/nn)	1.11
	HLA-DRB3 * 02:02	NetMHCIIpan	2.37
	HLA-DRB5 * 01:01	Consensus (smm/nn/sturniolo)	2.92
	HLA-DRB1 * 15:01	Consensus (smm/nn/sturniolo)	14.19
	HLA-DRB1 * 03:01	Consensus (smm/nn/sturniolo)	14.96
	HLA-DRB3 * 01:01	Consensus (comb.lib./smm/nn)	20.72
	HLA-DRB4 * 01:01	Consensus (comb.lib./smm/nn)	32.06
**Pep_SQ2FDVFYYLLTSASTPA (>ABV31248.1)**			
FDVFYYLLTSASTPA	HLA-DRB1 * 07:01	Consensus (comb.lib./smm/nn)	1.67
	HLA-DRB3 * 01:01	Consensus (smm/nn/sturniolo)	3.95
	HLA-DRB1 * 15:01	Consensus (smm/nn/sturniolo)	3.58
	HLA-DRB3 * 02:02	NetMHCIIpan	4.02
	HLA-DRB5 * 01:01	Consensus (smm/nn/sturniolo)	4.34
	HLA-DRB3 * 01:01	Consensus (comb.lib./smm/nn)	40.25
	HLA-DRB4 * 01:01	Consensus (comb.lib./smm/nn)	42.57
**Pep_PH3RAYALLFSKLGAAVV (PADG_08651)**			
	HLA-DRB5 * 01:01	Consensus (smm/nn/sturniolo)	3.19
	HLA-DRB1 * 15:01	Consensus (smm/nn/sturniolo)	3.37
	HLA-DRB1 * 07:01	Consensus (smm/nn/sturniolo)	4.79
	HLA-DRB3 * 02:02	NetMHCIIpan	11.93
	HLA-DRB1 * 03:01	Consensus (smm/nn/sturniolo)	17.05
	HLA-DRB4 * 01:01	Consensus (comb.lib./smm/nn)	23.65
	HLA-DRB3 * 01:01	Consensus (comb.lib./smm/nn)	32.36
**Pep_ SQ4ERVSIIANPAVASLY (PAAG_08203)**			
	HLA-DRB3 * 02:02	NetMHCIIpan	0.01
	HLA-DRB1 * 15:01	Consensus (smm/nn/sturniolo)	2.60
	HLA-DRB1 * 03:01	Consensus (smm/nn/sturniolo)	11.00
	HLA-DRB1 * 07:01	Consensus (comb.lib./smm/nn)	15.00
	HLA-DRB4 * 01:01	Consensus (comb.lib./smm/nn)	19.00
	HLA-DRB3 * 01:01	Consensus (comb.lib./smm/nn)	7.70
	HLA-DRB5 * 01:01	Consensus (comb.lib./smm/nn)	43.00

* HLA nomenclature.

**Table 4:** Prediction of the four epitopes found and ESAT-6 epitope in the IFNepitope algorithm.

Epitope	Method	IFN-ɤ	Score IFN-ɤ	Score Other Cytokines	Score Random IFN-ɤ
Epi 1—MSAFSRMTASLGFSK	Scan/MSV	Negative	−0.14	0.49	0.81
Epi 2—FDVFYYLLTSASTPA	Scan/MSV	Negative	−0.56	0.44	−0.00
Epi 3—RAYALLFSKLGAAVV	Scan/MSV	Positive	−0.16	0.61	0.00
Epi 4—ERVSIIANPAVASLY	Scan/MSV	Positive	0.15	0.51	−0.38
ESAT-6—QWNFAGIEAAASAIQ	San/MSV	Positive	0.30	0.88	0.63

## Supplementary Materials

The following are available online at https://www.mdpi.com/2309-608X/6/4/379/s1, Figure S1: Analysis of the interaction of the ESAT-6 protein by integrating different algorithm searches. (**A**) Signal peptide prediction (SignalP peptide). (**B**). Prediction of transmembrane regions (TMHMM). (**C**). Prediction of epitopic regions for MHC II (NetMHCII).
