# Supplementary material for: Prediction of Conserved Peptides of Paracoccidioides for Interferon-γ Release Assay: The First Step in the Development of a Lab-Based Approach for Immunological Assessment during Antifungal Therapy
Source: J Fungi (Basel). 2020 Dec 19;6(4):379. doi: 10.3390/jof6040379 (PMC7766394; doi:10.3390/jof6040379)
Supplement: Supplementary file 1 [file jof-06-00379-s001.pdf]

Supplementary Materials

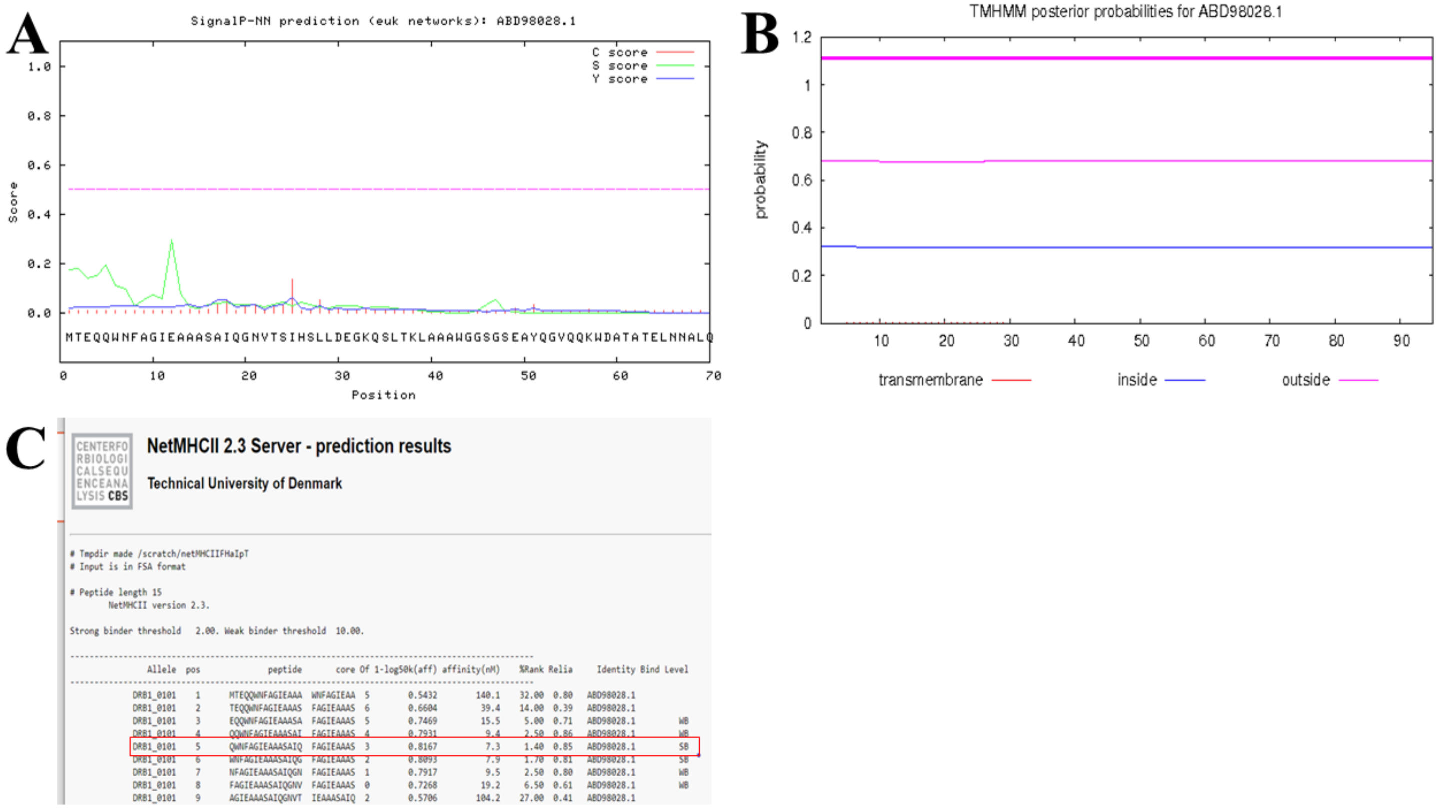

Figure S1. Analysis of the interaction of the ESAT-6 protein by integrating different algorithm searches. (**A**) Signal peptide prediction (SignalP peptide). (**B**). Prediction of transmembrane regions (TMHMM). (**C**). Prediction of epitopic regions for MHC II (NetMHCII).
